# Non-Monotonic Aerosol Effect on Precipitation in Convective Clouds over Tropical Oceans

**DOI:** 10.1038/s41598-019-44284-2

**Published:** 2019-05-24

**Authors:** Huan Liu, Jianping Guo, Ilan Koren, Orit Altaratz, Guy Dagan, Yuan Wang, Jonathan H. Jiang, Panmao Zhai, Yuk L. Yung

**Affiliations:** 1 State Key Laboratory of Severe Weather, Chinese Academy of Meteorological Sciences, Beijing, 100081 China; 2 College of Earth and Planetary Sciences, University of Chinese Academy of Sciences, Beijing, 100049 China; 3 Department of Earth and Planetary Sciences, Weizmann Institute of Science, Rehovot, 76100 Israel; 4 Division of Geological and Planetary Sciences, California Institute of Technology, Pasadena, CA 91125 USA; 5 Jet Propulsion Laboratory, California Institute of Technology, Pasadena, CA 91109 USA

**Keywords:** Environmental impact, Atmospheric science

## Abstract

Aerosol effects on convective clouds and associated precipitation constitute an important open-ended question in climate research. Previous studies have linked an increase in aerosol concentration to a delay in the onset of rain, invigorated clouds and stronger rain rates. Here, using observational data, we show that the aerosol effect on convective clouds shifts from invigoration to suppression with increasing aerosol optical depth. We explain this shift in trend (using a cloud model) as the result of a competition between two types of microphysical processes: cloud-core-based invigorating processes vs. peripheral suppressive processes. We show that the aerosol optical depth value that marks the shift between invigoration and suppression depends on the environmental thermodynamic conditions. These findings can aid in better parameterizing aerosol effects in climate models for the prediction of climate trends.

## Introduction

Convection in the rising tropical branch of the Hadley cell is known to play a key role in the global energy balance and water cycle, producing convective clouds with significant amounts of rain^1^. Aerosols—solid or liquid particles suspended in the atmosphere—affect the planetary energy balance by interacting directly with solar radiation and affecting cloud properties. A better understanding of aerosol effects on clouds is regarded as one of the most important and toughest challenges in climate research because the governing mechanisms and the overall effect are still not well understood^2^. Aerosols serve as cloud condensation nuclei and ice nuclei, affecting cloud microphysics and its coupling with dynamics^3–5^. Initially, a polluted cloud has a narrower distribution of smaller cloud droplets^6^. This initial change has been shown to affect a chain of microphysical and dynamic processes and thus the cloud’s macrophysical and optical properties, as well as rain production^7–11^. More aerosols activate more droplets that compete for available supersaturation. Although the droplets are smaller, their collective surface area increases and therefore diffusion efficiency increases^12,13^, yielding stronger latent heat flux that strengthens the cloud’s updraft. Collection processes are less efficient for smaller droplets, delaying the onset of rain^8,14^. Smaller droplets have greater mobility^15^ and can, therefore, be carried higher into the atmosphere by the stronger updrafts. A larger water mass is pushed above the freezing level, and smaller supercooled droplets freeze at colder temperatures^16^. Thus, the latent heat of freezing is released at higher levels, again boosting the updrafts^17^. All of these processes tend to yield deeper clouds that hold more water^9,18^; hence, once collection processes start, they are more efficient (also due to a larger contrast among hydrometeor sizes), thus implying faster collection that creates larger raindrops and larger overall rain yield^10^. However, driven by the same processes in the subsaturated areas of the clouds (usually within the margins), aerosols can act as a suppressive factor by enhancing evaporation sublimation, cooling and mixing^12–19^. The outcome of these competing effects depends on the thermodynamic conditions^20,21^ and the stage of the cloud’s lifetime. In addition to the internal effects on clouds, direct interaction of aerosols with solar radiation in cloud-free areas yields scattering and absorption, which may warm the aerosol layer and cool the atmosphere below it and the Earth’s surface, thereby stabilizing the lower atmosphere and suppressing convection and rain^22^. This complex system, which is structured by many competing effects that act both inside and outside of clouds, makes estimating the overall aerosol effect on convective clouds extremely challenging. This is demonstrated by previous studies on the link between cloud properties and aerosols, which have suggested no significant influence^23^, weak suppression^24^, or a boomerang trend (from invigoration to suppression)^11,25^.

Koren *et al*.^10^ showed a clear positive correlation between rain rate (R) and Aerosol Optical Depth (AOD), laying a solid foundation for the intensifying effect of aerosols on rain. Here, we explored this link for convective systems over tropical oceans with the aim of unifying and expanding previous studies. We used numerical modeling to suggest an underlying mechanism.

## Methods

Three databases were used over the tropical ocean during the summers of 2003–2012 (June, July, and August). The Moderate Resolution Imaging Spectroradiometer (MODIS) Aqua data were used for AOD^26^ and cloud properties^27^. R was obtained from the Tropical Rainfall Measuring Mission (TRMM) satellite data^28^, and meteorological information was obtained from the European Centre for Medium-Range Weather Forecasts (ECMWF) ERA-Interim dataset^29^ (see more details in the SI). To analyze simultaneous information of aerosol, cloud and rain properties and corresponding meteorological conditions, we projected all datasets to a time window of ±3 h around the Aqua passing time (1330 local time) and averaged them to a similar spatial scale of 1°^10^. To partially correct the inherent bias in AOD data towards less cloudy conditions (MODIS cannot retrieve aerosol and cloud properties at the same time and location), we interpolated the data to regions that were obscured by clouds (limited to a distance of one grid-square)^10^.

**Figure 1:**
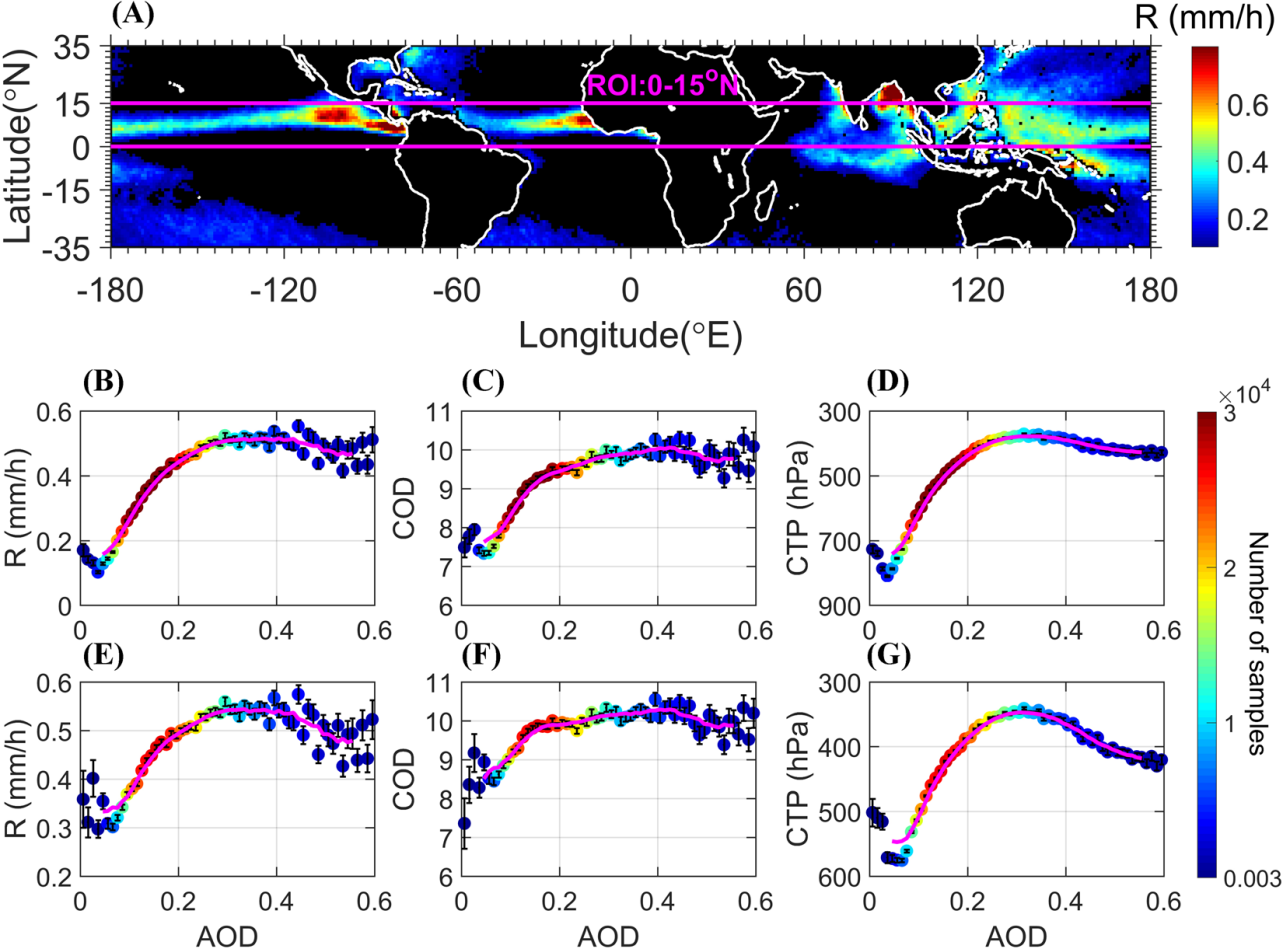
Map of the average R and associated plots of cloud properties as a function of the AOD (bin size of 0.01) at 1330 local time in JJA (2003–2012). (**A**) Average R for all days with AOD ≤ 0.6. The magenta lines mark the ROI. (**B**–**G**) For days with R > 0, and AOD ≤ 0.6 (**B**) R, (**C**) COD, and (**D**) CTP, while (**E**) R, (**F**) COD, and (**G**) CTP for days with R > 0, AOD ≤ 0.6, and CF ≥ 0.7. The colors denote the number of samples, the error bars represent the standard error, and the magenta curves are the corresponding 9-point moving averages.

We chose our region of interest (ROI, Fig. 1A) to cover the tropical ocean (0–15°N) because it is characterized by high-density of convective clouds that produce intense rain, and there is relatively small variability in the dynamic and thermodynamic conditions in this region during a specific season.

### Cloud-resolving model (TAU-CM)

The Tel Aviv University axisymmetric nonhydrostatic cloud model (TAU-CM), with the detailed treatment of cloud microphysics^30,31^, was used to explore the physical mechanisms underlying the observed relation of cloud and rain properties with aerosol loading.

Four hydrometeor species were considered: drops, ice crystals, graupel particles and aggregates (snowflakes). The liquid-phase microphysical processes treated by the model including drop nucleation, condensation and evaporation, collision–coalescence, break-up and sedimentation. The considered ice-phase processes were ice nucleation (deposition, condensation–freezing, contact nucleation, and immersion freezing), ice multiplication, deposition and sublimation of ice particles, ice–ice and ice–drop interactions (coagulation, accretion, or riming), melting of ice particles and sedimentation. The microphysical processes were formulated and solved using a multimoment bin method^32^. The background aerosol size distribution represented a clean maritime environment^33^. Ten different simulations were conducted for each initial atmospheric profile, simulating a wide range of aerosol loading conditions, from extremely pristine (total concentration of 5 cm^−3^) to polluted (10,000 cm^−3^)^12^.

The model resolution was set to 150 m in both the vertical and horizontal directions, with a time step of 1 s. Convection was initiated by a warm bubble near the bottom of the domain.

We used three different sets of initial environmental conditions based on idealized atmospheric profiles that characterize a moist tropical environment (Fig. S4). Each of the profiles included a well-mixed subcloud layer between 0 and ~900 m, a conditionally unstable cloud layer (6.5 °C/km) between 900 and 10,000 m, and an overlying inversion layer. Three different RH levels were used for the cloudy layer to represent different humidity conditions. The RH above the inversion layer was 30% in all profiles (see more details in the SI).

## Results

Figure 1A presents the average R for JJA (between 2003 and 2012), including all days with AOD ≤ 0.6. The tropical oceans (the ROI, as marked in the figure) can be easily recognized as a belt of intense rain (regions with average R < 0.1 mm/h were excluded from the analysis). Figure 1B–D shows the average R and the corresponding cloud properties (Cloud Optical Depth: COD; Cloud Top Pressure: CTP) as a function of the AOD (averaged into 60 equal AOD-range bins). All panels show a nonmonotonic relationship with an increase in cloud depth and rain intensity as the AOD increases in the low AOD regime (relatively clean environment), followed by a decrease for the high-end AOD values. We defined the optimal AOD value (AOD_op_) as that corresponding to the maximum R (0.3–0.4 in this case; see Fig. 1B). This value marks the turning point from invigoration to suppression in the trend. Such nonmonotonic dependence suggests competition between at least two dominant processes^34,35^. However, before investigating aerosol-related processes, we explored the possible influence of meteorology on both aerosols and clouds that can produce apparent correlations with no real causality^36^. We inspected the changes in AOD and R with the most relevant thermodynamic variables estimated using reanalysis data [ERA-Interim^29^]. The vertical pressure velocity at 400 hPa (ω_400_; ~7 km; in –Pa/s, with negative and positive values representing downdrafts and updrafts, respectively) and Relative Humidity at 500 hPa (RH_500_; ~5.5 km) were shown previously to affect convective clouds and have the highest correlations with R^10^.

**Figure 2:**
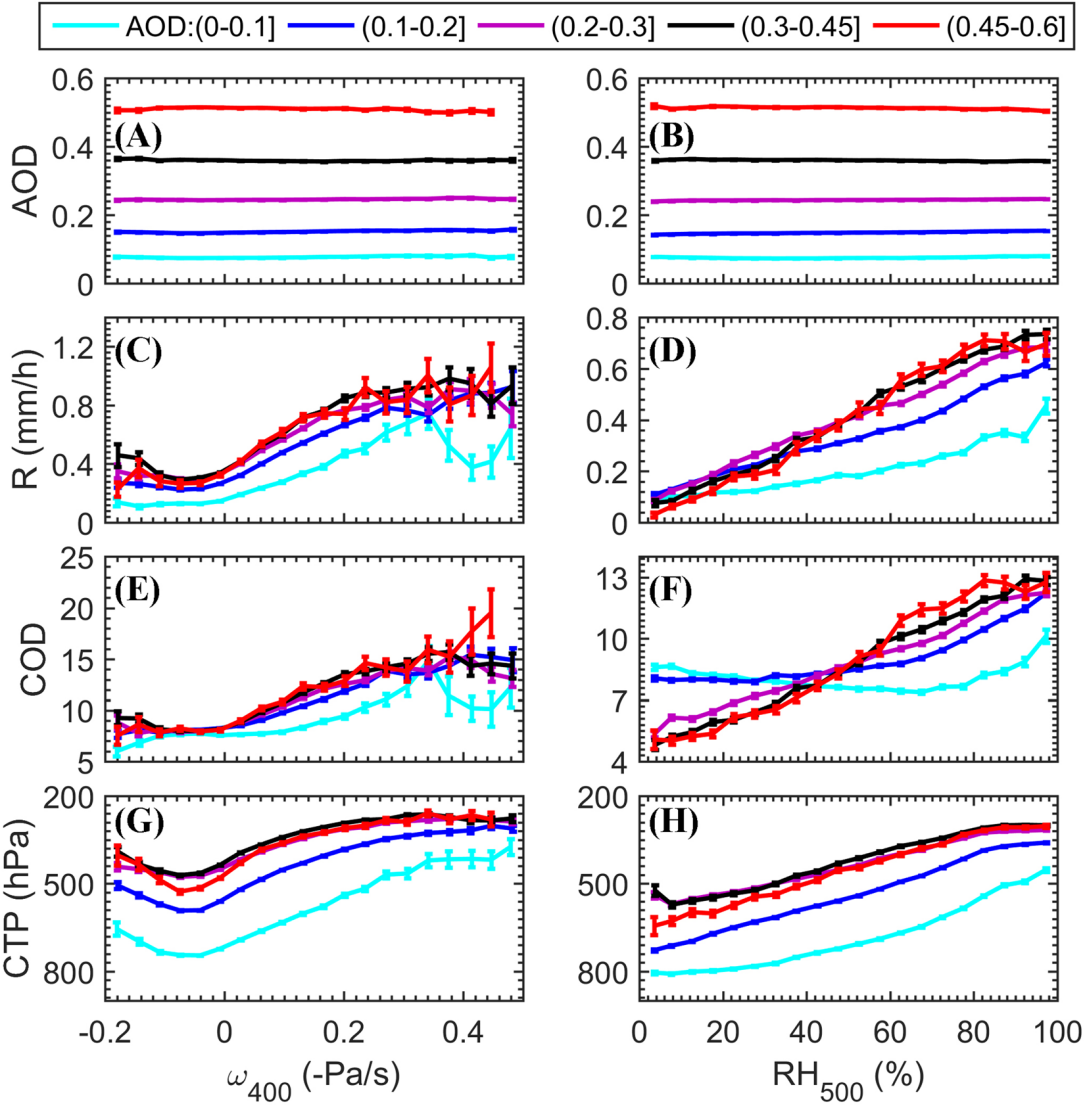
AOD and cloud properties as a function of two meteorological parameters for five AOD levels, over the ROI at 1330 local time in JJA (2003–2012). (**A**) AOD versus ω_400_ (bin size of 0.14 Pa/s), (**B**) AOD versus RH_500_ (bin size of 5%), (**C**) R versus ω_400_, (**D**) R versus RH_500_, (**E**) COD versus ω_400_, (**F**) COD versus RH_500_, (**G**) CTP versus ω_400_, and (**H**) CTP versus RH_500_. The error bars represent standard errors.

All data were divided into five AOD subgroups, then sorted by ω_400_ (Fig. 2, left panels) and RH_500_ separately, and averaged into 20 equal ω_400_ or RH_500_ bins. The analysis of AOD as a function of these two meteorological parameters (Fig. 2A,B) shows almost straight lines, meaning no significant dependence between them. Stronger updrafts and a higher RH in the upper troposphere imply favorable conditions for convective cloud development, and indeed, Fig. 2C–H show stronger R and deeper clouds under such conditions. The nonmonotonic trend with AOD “survives” the meteorological slicing along most of the meteorological regime spanned by the two selected variables. When slicing the data per key meteorological parameter (for example, per given ω_400_ value, say 0.2 -Pa/s), the five AOD subgroups show a nonmonotonic response of R, COD and CTP to the increase in AOD, for which the AOD_op_ (representing the maximum cloud or rain value per given meteorological parameter) is not the highest AOD in most cases.

What aerosol effects might explain the observed trend? The increasing branch is likely to be linked to the aerosol invigoration effect, in which more but smaller activated droplets condense water more efficiently and enhance the updraft by releasing more latent heat, elevating the smaller droplets (that have a smaller effective terminal velocity) higher in the cloud in which freezing will occur at lower temperatures, which further invigorates convection^3–5^. A Delay in the onset of precipitation processes in deeper clouds that contain more condensate yields stronger rain^10^. For the decreasing branch and the nonmonotonic trend in cloud properties with AOD, this trend has been shown over the Amazon^25^. The decreasing branch has been attributed to the absorption effect of aerosols. The study showed that significant extinction of direct solar radiation is required to warm the aerosol layer by absorption. The absorption efficiency is therefore a strong function of cloud coverage. For highly cloudy conditions (close to overcast), aerosol absorption is unlikely to have a dominant effect. To verify this, we analyzed a limited subset of the data (Fig. 1E–G, Fig. S3) characterized by a high cloud fraction (CF > 0.7). The results show a similar nonmonotonic trend, suggesting that the radiative effect is likely to be less important in our case. Moreover, aerosol radiative effects are likely to be weaker over the ocean since the ocean’s heat capacity ensures that the surface temperature does not change rapidly. Note that the dataset for days with R > 0, AOD ≤ 0.6, and CF ≥ 0.7 was used for further analysis (the distributions are shown in the SI, Fig. S3). Geographical shifts in sampling and wet scavenging^37^ were analyzed as well (see SI, Figs S1, S2), but were not found as possible mechanisms behind the observed trends.

**Figure 3:**
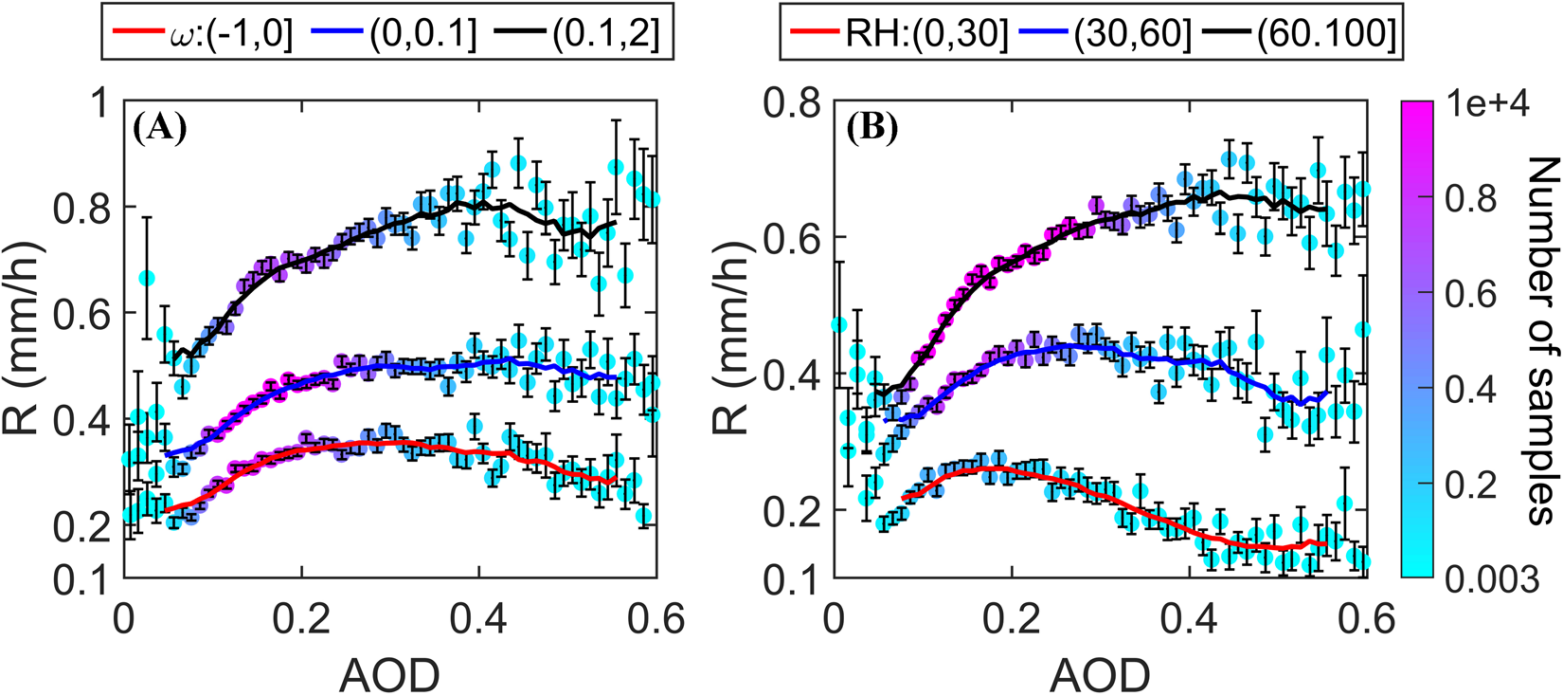
R as a function of AOD (bin size of 0.01) for specific meteorological conditions over the ROI at 1330 local time in JJA (2003–2012). (**A**) For ω_400_ ranges; (**B**) for RH_500_ ranges. The colors show the number of samples per bin, the error bars represent standard errors, and the red, blue and black lines represent their corresponding 9-point moving average curves.

The results shown in Figs 2 and S2 suggest that the AOD_op_ depends on the ambient thermodynamic conditions. To further explore this, we sliced ω_400_ and RH_500_ into three specified ranges and explored the associations between R and AOD per meteorological condition subset. Figure 3 shows an increase in AOD_op_ with increasing RH_500_ and ω_400_, suggesting that the invigoration branch extends to higher AOD levels under more humid and unstable conditions, which normally corresponds to deeper clouds. A similar shift towards higher values of AOD_op_ can be seen in Fig. S2 for increased rain rates.

**Figure 4:**
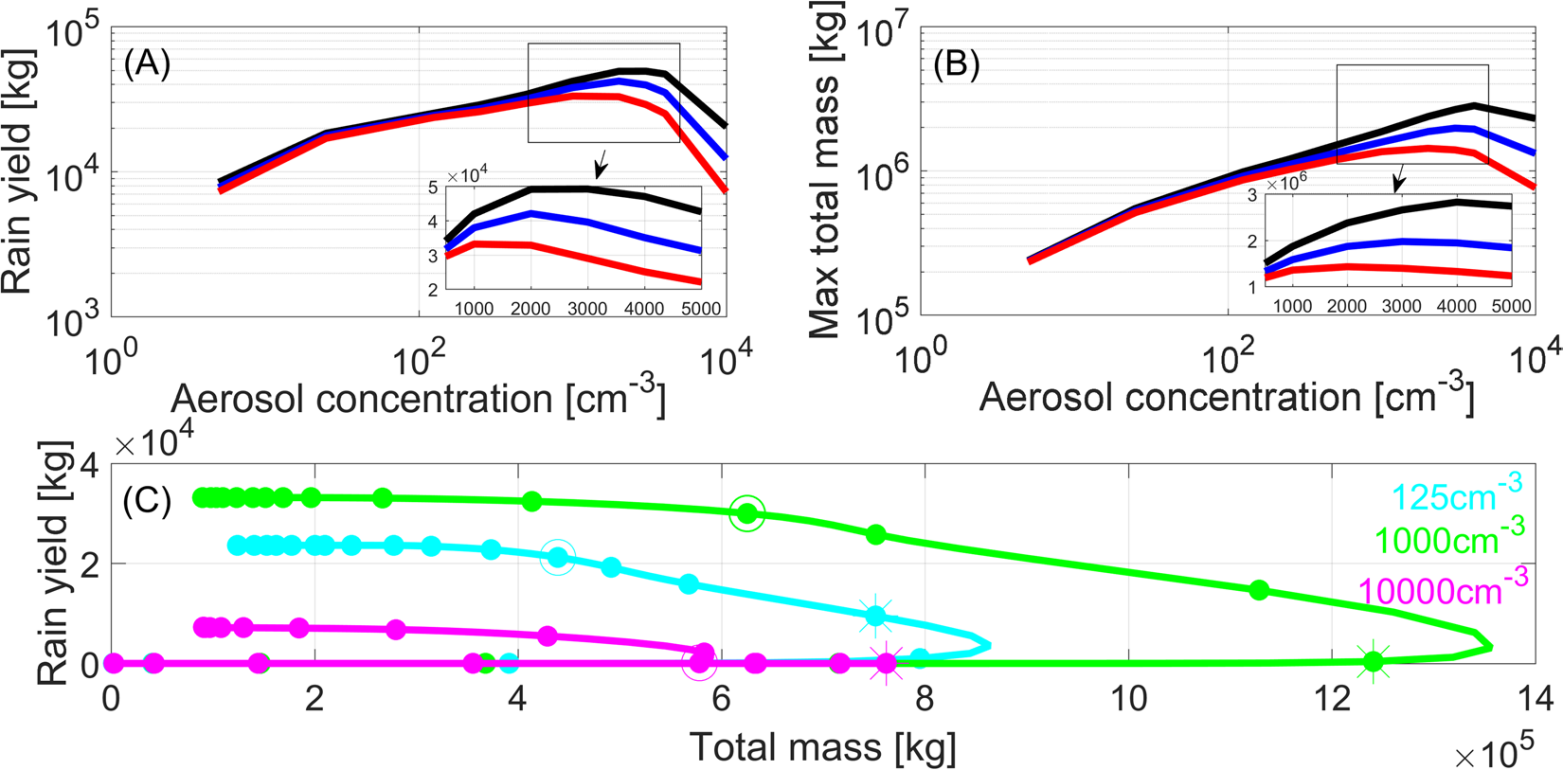
Numerical cloud simulation results. (**A**) Total surface rain yield per simulated cloud and (**B**) cloud’s maximum total mass as a function of aerosol concentration used in the simulation. Note that each curve represents 10 simulations conducted using the same atmospheric profile; the red, blue, and black lines represent a gradually more humid environment (RH_500_ of 50.8, 63.3, and 75.8%, respectively; see Fig. S4 for the initialization profiles). The insets show a zoomed-in view of the boxed parts of the curves. (**C**) Temporal evolution of the total cloud mass versus rain yield for three clouds formed under the driest conditions (RH_500_ = 50.8%, red profile in Fig. S4) with different aerosol concentrations (125, 1000, and 10,000 cm^−3^). Note that the dots represent 5-min intervals, and the stars and circles represent simulation times of 30 and 45 min, respectively.

Following the observed trends, as presented above, we ran a set of bin-microphysics simulations of single deep convective clouds (TAU-CM; see details in the methods section) to explore a possible underlying mechanism. The numerical experiments were based on three sets of initial thermodynamic conditions that differed in their humidity profiles (Fig. S4), all representing idealized atmospheric profiles of a moist tropical environment. For each thermodynamic profile, 10 runs were conducted with different aerosol concentrations (from 5 to 10,000 cm^−3^ near ground level). Figure 4A,B present the total surface rain yield and maximum total mass, respectively, for each simulation (per given set of initial conditions; RH_500_ = 50.8, 63.3, and 75.8%, Fig. S4) as a function of the aerosol concentration. Similar to the observational analysis results, a reversed trend is shown for both total rain yield and maximum cloud mass with increasing aerosol concentration. Moreover, as seen in Fig. 4A,B the simulated optimum in the aerosol concentration values (which is an analogue quantity to AOD_op_ that is determined in the observational analysis) are larger for the more humid simulated profiles (for both total rain yield and maximum mass).

To better describe the competing effects among cloud processes, we followed the temporal evolutions of pristine, polluted and extremely polluted clouds (125, 1000, and 10,000 cm^−3^, respectively) in the phase space spanned by total mass versus rain yield. This phase space enables examination of the aerosol effects on the production of total cloud mass versus rain yield based on the differences in the trajectories of the three clouds. The same initial thermodynamic profile was used for the three runs with RH_500_ = 50.8% (red line in Fig. S4). The delay in precipitation onset with increasing aerosol concentration is very clear (as seen by the different timing of the vertical shifts in the curves). In the clean case (cyan curve: 125 cm^−3^), the initial rainfall at the surface (after ~20 min of simulation) occurs before the total mass reaches its maximum value (maximum value along the X axis). The rain process starts early, in the warm part of the cloud and is driven by an efficient collection process. The early rain yield limits the cloud’s development and therefore the total rain amount. For the case with intermediate-level pollution (green curve, 1000 cm^−3^), the ground precipitation starts at ~30 min, after the cloud has developed into a mixed-phase cloud, with falling graupel particles being the main source. In this case, the cloud develops for a longer time, and the precipitation particles form mainly by riming a larger amount of supercooled water on ice particles. Moreover, stronger updrafts and better droplet mobility transfer more mass higher into the atmosphere. Hence, the cloud is deeper, and the maximum total cloud mass is significantly larger than it is in the clean cloud case. In the extremely polluted case (magenta curve, 10,000 cm^−3^), precipitation initiates ~45 min into the simulation after the cloud’s total mass has reached its maximum value, which occurs much earlier (30 min). This implies that mixing and entrainment processes have enough time to enhance the evaporation and sublimation of hydrometeors at the cloud’s margin and deplete the cloud’s water mass. In this cloud, the rain process is also driven by graupel formation, but it is delayed compared to the intermediate-level polluted cloud. Cloud depletion by entrainment processes before the onset of significant rain processes leaves less condensate and therefore reduces rain yield.

The cloud that produced a larger water content also produced a larger amount of rain, and this case corresponds to the conditions of optimal aerosol concentration. These simulations demonstrate how ambient RH controls the balance between the net generation and the net loss of condensate mass. An increase in RH can dramatically reduce evaporation and sublimation processes, leading to an increase in AOD_op_ value.

## Discussion

Using observational and reanalysis data, we show a nonmonotonic trend in convective cloud properties and rain intensity as a function of aerosol loading. The detailed structure of the trend, specifically the AOD_op_ for which cloud and rain properties reach their maximum value, depends on the ambient thermodynamic conditions. With the aid of a cloud model, we suggest an explanation of this trend as a result of competing effects: cloud-core-based processes that act to invigorate clouds and therefore amplify rain versus cloud-periphery processes that act to mix clouds with drier air, which enhances evaporation and dissipation. The AOD_op_ value is higher under more unstable conditions, in which the cloud-core processes dominate, and under humid conditions, in which entrainment is weaker. This study extends a previous work that focused only on the invigorating branch^10^ and a modeling study^20^ that focused on warm clouds, showing a reversal trend and similar links between AOD_op_ and thermodynamics. Moreover, this study unifies some of the conclusions obtained in previous works highlighting the central role of RH in the cloudy layer in controlling aerosol effects on clouds and rain, and the competition between condensational heating and evaporative cooling for the total aerosol effect^21^.

A better understanding of the link between convective clouds and aerosols and their dependence on environmental properties will yield better parameterizations of clouds in climate models and better climate predictions.

## Supplementary information

Supplementary information

## Data Availability

All observational datasets used in this study are publicly available. The numerical results are available from the corresponding author upon request.
