# Supplementary material for: Non-Monotonic Aerosol Effect on Precipitation in Convective Clouds over Tropical Oceans
Source: Sci Rep. 2019 May 24;9:7809. doi: 10.1038/s41598-019-44284-2 (PMC6534586; doi:10.1038/s41598-019-44284-2)
Supplement: Supplementary file 1 — Supplementary information [file 41598_2019_44284_MOESM1_ESM.pdf]

Supplementary Materials for

Non-Monotonic Aerosol Effect on Precipitation in Convective Clouds over Tropical Oceans

Huan Liu[1,2,3], Jianping Guo[1*], Ilan Koren[3*], Orit Altaratz[3], Guy Dagan[3], Yuan Wang[4], Jonathan H. Jiang[5], Panmao Zhai[1], and Yuk L. Yung[4]

Correspondence to: J.P. Guo (jpguocams@gmail.com); I. Koren(ilan.koren@weizmann.ac.il)

This PDF file includes:

Supplementary Text

Figures S1 to S4

Table S1

Reference List

Supplementary Text

Observational datasets

(1) AOD[1] (550 nm) and cloud properties[2] (Cloud Optical Depth: COD; Cloud Top Pressure: CTP; and Cloud Fraction: CF) measurements were taken from the Moderate Resolution Imaging Spectroradiometer (MODIS), Level 3 version 6.1 onboard the Aqua satellite, with a 1° spatial resolution.

(2) R measurements were taken from the Tropical Rainfall Measuring Mission (TRMM) satellite 3B42 version 7 product with a 0.25° spatial resolution[3].

(3) Meteorological information was taken from the European Centre for Medium-Range Weather Forecasts (ECMWF) ERA-Interim dataset (the vertical pressure velocity at 400 hPa: ω_{400}; Relative Humidity at 500 hPa: RH_{500}) with a 1° spatial resolution[4].

We restricted the AOD values to ≤0.6 to minimize cloud contamination[5] and excluded all statistics with less than 30 samples (days) to guarantee the validity of our analyses[6].

The results shown in the paper are for June, July, and August (Figs. S1-S3) to exclude possible seasonal variations.

Cloud-resolving model (TAU-CM) and its setup

A set of prognostic equations were solved by the model for the vertical and radial velocities, pressure perturbation, virtual potential-temperature perturbation, specific-humidity perturbation, specific number concentration, aerosol mass in a spectral bin, specific number concentration, mass for each type of cloud particle in a size bin, and the concentration of activated ice nuclei[7,8].

Each spectrum of cloud particles was divided into 34 bins with mass doubling for adjacent bin boundaries. The liquid-phase microphysical processes treated by the model included drop nucleation, condensation and evaporation, collision–coalescence, break-up and sedimentation. The considered ice-phase processes were ice nucleation (deposition, condensation–freezing, contact nucleation, and immersion freezing), ice multiplication, deposition and sublimation of ice particles, ice–ice and ice–drop interactions (coagulation, accretion, or riming), melting of ice particles and sedimentation. The microphysical processes were formulated and solved using a multimoment bin method[9].

Ten different simulations were conducted for each initial atmospheric profile, with total aerosol concentrations of 5, 25, 125, 250, 500, 1000, 2000, 3000, 4000 and 10,000 cm^{-3} (near ground level)[10]. The aerosol concentration was assumed to decrease exponentially with height, with an e-fold of 2000 m. To reduce the results' sensitivity to the shape of the aerosol size distribution and to focus on the aerosol number concentration effect, the different aerosol

concentrations were calculated by multiplying all bins by a constant factor and maintaining a constant shape of the size distribution. To avoid the giant cloud-condensation nuclei (GCCN) effect, the aerosol size distribution was truncated at 1μm.

Geographical distribution

The geographical distribution of local R-AOD correlations is analyzed here. Fig. S1 shows R differences (polluted-clean) for two AOD subsets: below 0.3 (corresponding to the increasing branch) and above 0.3 (the decreasing branch). Here we divided each subset into three equal groups, with the lowest-third AOD days labeled as clean and the highest third AOD days labeled as polluted. Nonmonotonic relationships between R and AOD are observed, suggesting no significant influence of geographical shifts in sampling on the observed trend.

R vs. AOD for subsets of rain rate

To better understand the R-AOD nonmonotonic correlation, we performed a similar analysis to that performed in Fig. 1B but for four different R subsets: $0<R\leq0.01$ mm/h, $0.01<R\leq0.1$ mm/h, $0.1<R\leq1$ mm/h, and $R>1$ mm/h. Interestingly, Fig. S2A shows a monotonic negative correlation between R and AOD; Fig. S2B-C shows nonmonotonic R-AOD correlations; while Fig. S2D shows monotonic positive correlation between R and AOD. These results again demonstrate the increase in AOD_{op} value for systems with stronger rain intensity, and they also suggest no significant wet scavenging effect[11].

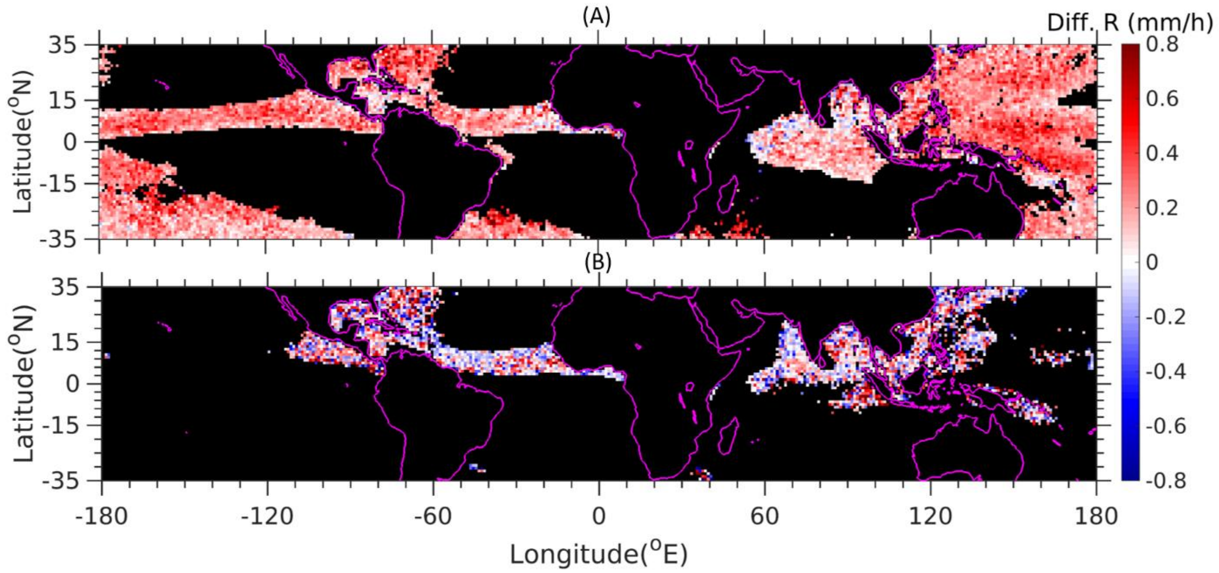

Fig. S1.

Maps of R differences (polluted-clean) at 1330 local time in JJA (2003-2012). (A) 0<AOD≤0.3; (B) 0.3<AOD≤0.6. The polluted/clean set is defined as days with the highest/lowest 1/3 of AODs and R>0. The results for grid cells with less than 30 polluted/clean samples were excluded.

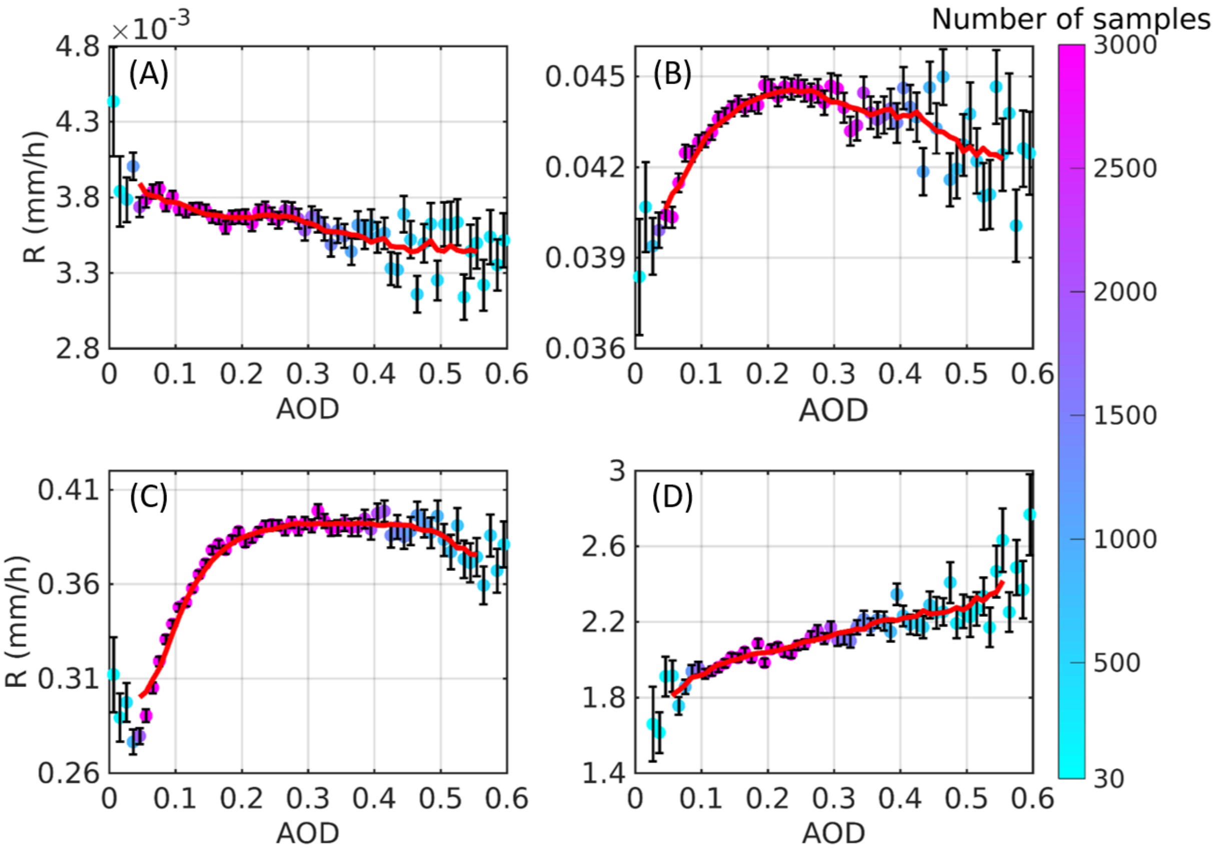

Fig. S2.

The same as Fig. 1B but for four R subgroups. (A) 0<R≤0.01 mm/h; (B) 0.01<R≤0.1 mm/h; (C) 0.1<R≤1 mm/h; (D) R＞1 mm/h.

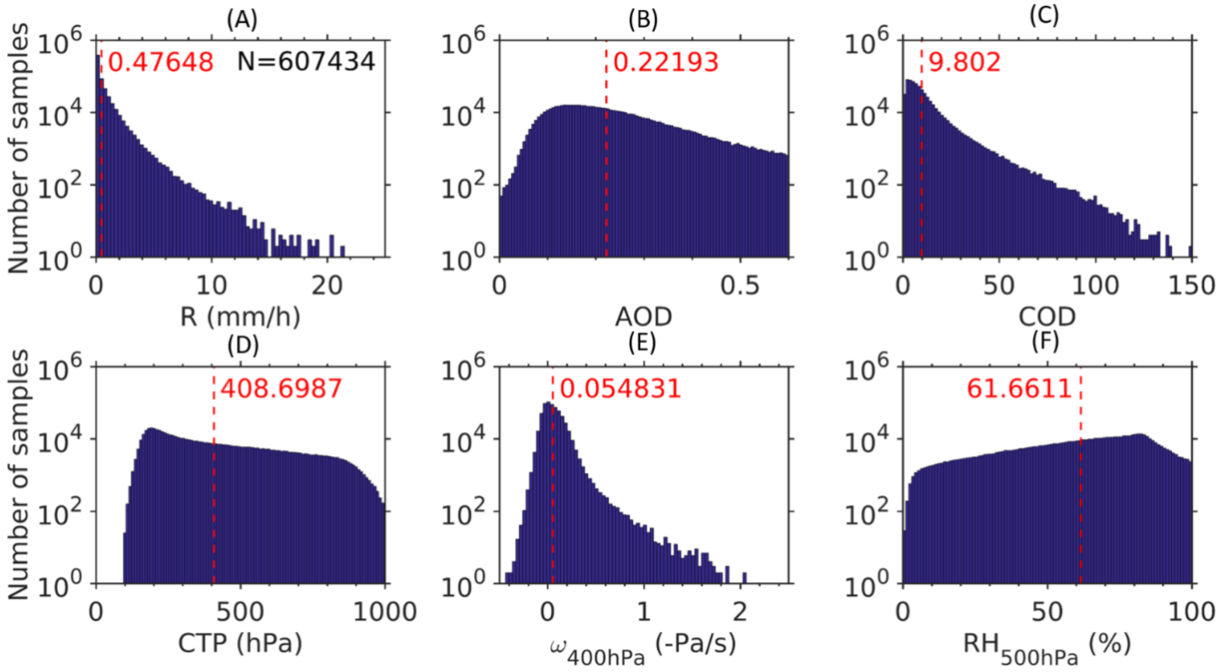

Fig. S3.

Histograms of the dataset with R>0, AOD≤0.6, and CF≥0.7 over the ROI. The red lines and numbers mark the average values of each factor.

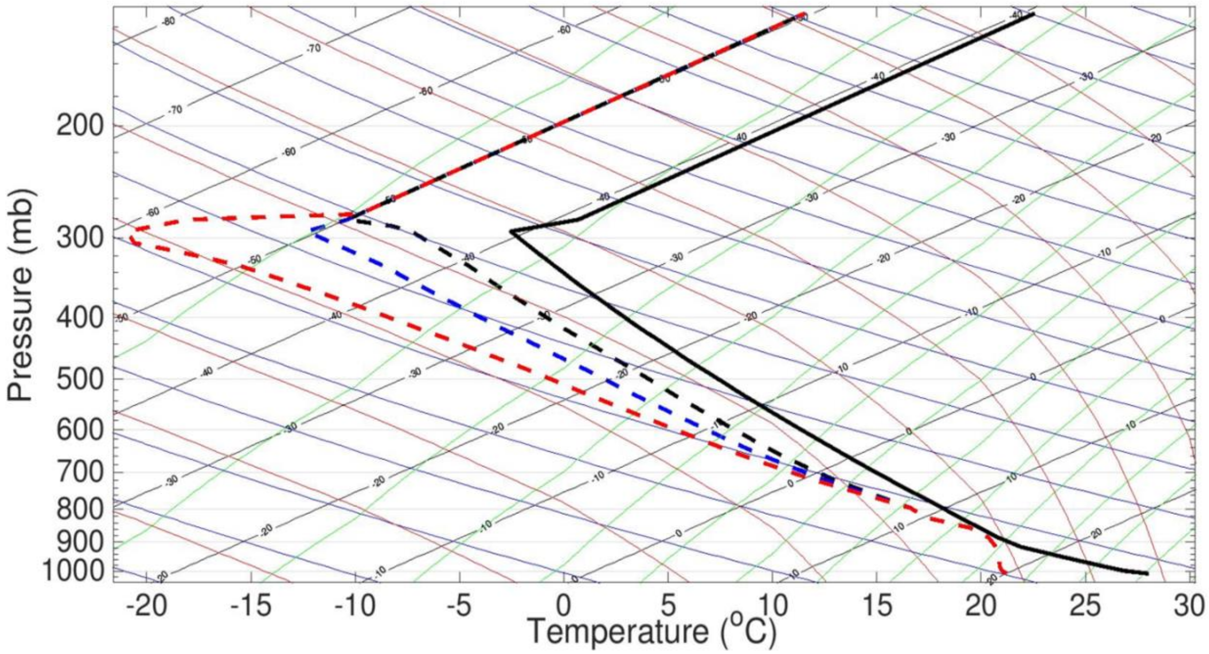

Fig. S4.

Thermodynamic diagram that presents the three initial atmospheric profiles. The black solid line denotes the temperature profile, and the dashed lines denote the dew-point temperature (red, blue, and black dashed lines correspond to the red, blue, and black lines in Fig. 4A and B; the red dashed line represents the profile used for Fig. 4C).

Table S1.

Data specifications used in this study: TRMM-retrieved precipitation, Aqua-retrieved AOD and cloud properties, and ECMWF reanalysis of meteorological data during June, July, and August (JJA) between 2003 and 2012.

**Source**		**Data**	**Horizontal resolution (°)**	**Temporal resolution**
TRMM	3B42, V7	Precipitation	0.25°×0.25°	3-hourly
MODIS	MYD08-D3, V6.1	AOD (550nm)	1°×1°	Daily
		COD	1°×1°	Daily
		CTP	1°×1°	Daily
		CF	1°×1°	Daily
ECMWF	ERA-Interim	Vertical velocity (400hPa)	1º×1º	6-hourly
		Relative humidity (500hPa)	1º×1º	6-hourly

Reference List

1. Levy, R. C., Mattoo, S., Munchak, L. A., Remer, L. A., Sayer, A. M., Patadia, F. & Hsu, N. C. The Collection 6 MODIS aerosol products over land and ocean. *Atmos. Meas. Tech.* ***6***, 2989-3034 (2013).

2. Platnick, S., Meyer, K. G., King, M. D., Wind, G., Amarasinghe, N., Marchant, B., Arnold, G. T., Zhang, Z. B., Hubanks, P. A., Holz, R. E., Yang, P., Ridgway, W. L. & Riedi, J. The MODIS cloud optical and microphysical products: collection 6 updates and examples from Terra and Aqua. *IEEE Trans. Geosci. Remote Sensing.* ***55***, 502-525 (2017).

3. Huffman, G. J., Adler, R. F., Bolvin, D. T., Gu, G. J., Nelkin, E. J., Bowman, K. P., Hong, Y., Stocker, E. F. & Wolff, D. B. The TRMM multisatellite precipitation analysis (TMPA): quasi-global, multiyear, combined-sensor precipitation estimates at fine scales. *J. Hydrometeorol.* ***8***, 38-55 (2007).

4. Dee, D. P., Uppala, S. M., Simmons, A. J., Berrisford, P., Poli, P., Kobayashi, S., Andrae, U., Balmaseda, M. A., Balsamo, G., Bauer, P., Bechtold, P., Beljaars, A. C. M., van de Berg, L., Bidlot, J., Bormann, N., Delsol, C., Dragani, R., Fuentes, M., Geer, A. J., Haimberger, L., Healy, S. B., Hersbach, H., Hólm, E. V., Isaksen, L., Kållberg, P., Köhler, M., Matricardi, M., McNally, A. P., Monge-Sanz, B. M., Morcrette, J. J., Park, B. K., Peubey, C., de Rosnay, P., Tavolato, C., Thépaut, J. N. & Vitart, F. The ERA-Interim reanalysis: Configuration and performance of the data assimilation system. *Q. J. R. Meteorol. Soc.* ***137***, 553-597 (2011).

5. Brennan, J. I., Kaufman, Y. J., Koren, I. & Li, R. R. Aerosol-cloud interaction-misclassification of MODIS clouds in heavy aerosol. *IEEE Trans. Geosci. Remote Sensing*. ***43***, 911-915 (2005).

6. Wilks, D. S. Statistical Methods in the Atmospheric Sciences. 3rd ed. Oxford: Academic press, 704 (2011).

7. Reisin, T., Levin, Z. & Tzivion, S. Rain production in convective clouds as simulated in an axisymmetric model with detailed microphysics. Part I: Description of the model. *J. Atmos. Sci.* ***53***, 497-519 (1996).

8. Yin, Y., Carslaw, K. & Feingold, G. Vertical transport and processing of aerosols in a mixed-phase convective cloud and the feedback on cloud development. *Q. J. R. Meteorol. Soc.* ***131***, 221-245 (2005).

9. Tzivion, S., Feingold, G. & Levin, Z. An efficient numerical solution to the stochastic collection equation. *J. Atmos. Sci.* ***44***, 3139-3149 (1987).

10. Dagan, G., Koren, I. & Altaratz O. Competition between core and periphery-based processes in warm convective clouds–from invigoration to suppression. *Atmos. Chem. Phys.* ***15***, 2749-2760 (2015).

11. Yoo, J. M., Lee, Y. R., Kim, D., Jeong, M. J., Stockwell, W. R., Kundu, P. K., Oh, S. M., Shin, D. B. & Lee, S. J. New indices for wet scavenging of air pollutants (O_3, CO, NO_2, SO_2, and PM_{10}) by summertime rain. *Atmospheric Environ.* **82**, 226-237 (2014).
